# Delayed Presentation of Antipsychotic Withdrawal Tardive Dyskinesia: A Case Report

**DOI:** 10.7759/cureus.43693

**Published:** 2023-08-18

**Authors:** Siddhant Solanki, Lakshmi Sai Deepak Reddy Velugoti

**Affiliations:** 1 Department of Medicine, Tbilisi State Medical University, Tbilisi, GEO; 2 Department of Research, California Institute of Behavioral Neurosciences and Psychology, Fairfield, USA

**Keywords:** #tardive dyskinesia, #withdrawal dyskinesia, #anti-psychotics, #risperidone, #dopamine receptor blocking agent

## Abstract

Tardive dyskinesia (TD) is an iatrogenic cause that occurs after prolonged use of antipsychotic and other dopamine-related medications and is described as repetitive, involuntary movement of muscles in the body. This case report focuses on an 80-year-old man who presented with a medical history encompassing dementia, bilateral blindness, glaucoma, hearing loss, hypertension, and hypercholesterolemia. He visited the clinic with a complaint of experiencing visual hallucinations. Investigations, including a CT head and EEG, revealed normal results, and Risperidone was prescribed. In the subsequent visit, Risperidone was prescribed again with Quetiapine and then replaced with valproate because of no change in symptoms and medication non-adherence. Over time, the patient felt better but started to have orofacial tremors after three months of discontinuing antipsychotic medications. For the orofacial tremors, the patient was prescribed Duetetrabenazine and asked for a follow-up visit. Early recognition and treatment of TD would be beneficial for these patients, and when prescribing, it's important to be aware that they may lead to delayed onset of TD.

## Introduction

Tardive dyskinesia (TD) is an iatrogenic disorder that occurs due to the long-term use of neuroleptic drugs in case of psychiatric disorders or anti-Parkinsonian or antiemetic medications [1]. It is described as the repetitive, involuntary movement of muscles in the body. Although the exact mechanism is unknown, multiple hypotheses explain the cause of TD. Among these, a prominent explanation is the heightened sensitivity of dopamine receptors resulting from prolonged use of anti-dopaminergic medications (chronic dopamine blockade) [2]. This mechanism explains the increase in the amplitude of TD with the reduction in the dosage of anti-dopaminergic medications and vice versa. TD is associated with risk factors such as age, gender, diagnosis, brain injury, the dosage of the causative drug, duration of the drug use, type of neuroleptic use, and concurrent use of multiple neuroleptic drugs [3]. TD is less common in childhood and more reversible, and females are more prone to develop TD with the exact dosage than men [4]. Studies showed that patients with schizophrenia are more prone to develop TD. Some studies have shown a positive correlation between dosage, duration of the use of drugs, and TD. Usually, patients using high-potency neuroleptics have more chances of developing TD than low-potency neuroleptics. Here we report an 80-year-old patient who was on Risperidone and Quetiapine for a long time and developed TD six months after stopping the medication.

## Case presentation

An 80-year-old man presented for evaluation of visual hallucinations for the first time in August 2021. The patient's medical history is significant for dementia, bilateral blindness, glaucoma, syncope, collapse, COVID-19, hearing loss, hypertension, and hypercholesterolemia. The patient has been experiencing intermittent visual hallucinations for over a year, seeing animals and people walking around the house. He reports poor sleep quality with frequent waking at night, and hallucinations are more pronounced in the evening and at night but also occur during the day. The patient underwent a workup, including a CT head, which was negative, and an EEG, which was normal. On examination, the patient presents with slow speech, preserved language function, and mild tremors without cogwheel rigidity or significant bradykinesia. He was on Risperidone 2 mg for several years before he came to us, and we added Quetiapine 50 mg in June 2022 to treat his hallucinations. However, the patient was noncompliant with Quetiapine for a month and had a recurrence of hallucinations. We resumed Quetiapine, but the patient continued to have visual hallucinations. On examination, the patient had a weight loss of 20 pounds, intermittent tremors, orofacial twitching, and gait disturbances. Both medications were stopped in August 2022, and a trial of Valproate 500 mg was initiated. Initially, visual hallucinations were under control, but later the patient was noncompliant, and Valproate levels were shown to be undetectable upon laboratory testing. The patient was advised to be compliant with medication. In March 2023, the patient presented with rapid, involuntary, repetitive tongue and jaw movements, which are depicted in Video 1 (written informed consent was obtained from the patient for the video). 

**Video 1:** Clinical presentation of Tardive dyskinesia (written informed consent was obtained from the patient for the video).

A trial of Duetetrabenazine 6 mg was given, and the patient's symptoms were controlled but not resolved, so he was advised on medication compliance and further follow-up.

## Discussion

In the case described, we made some significant observations. First, the patient experienced no tremors before using Risperidone and Quetiapine. Second, after stopping antipsychotic medication, it took about eight months for characteristic TD to manifest. The patient has often neglected to take his medications, and there was no alternative drug on his medication list that could cause his orofacial tremors.

The term *TD *is used to describe involuntary athetoid or choreiform movements (lasting at least a few weeks), usually of the tongue, lower face and jaw, and extremities (but occasionally involving the pharyngeal, diaphragmatic, or trunk muscles) developing in association with the use of a neuroleptic medication [5] for at least a few months. However, the most recent definition of TD is more inclusive and lists tardive dystonia as a subtype of TD syndrome [6].

The cause of TD is still unknown, but many theories have been put forth, including the following: (1) hypersensitivity of dopamine-2 (D2) receptor; (2) prolonged antipsychotic use-induced oxidative stress; (3) dysfunctional striatal gamma-aminobutyric acid (GABA) input to motor neurons; (4) reduced serotonin (5-hydroxytryptamine receptor 2A, or 5-HT2A) receptors expression; (5) general genetic susceptibility; and (6) lack of antipsychotic-metabolizing enzymes [7].

 In this case, the patient had known risk factors for TD, including advanced age, African ancestry, a longer length of disease, a history of antipsychotic use, and early Parkinsonism [3,8]. As tongue protrusion is a recognized indicator of TD [7], symptoms of TD can develop quickly after starting antipsychotic medication, especially in elderly patients. It is well-recognized that TD-like movements can develop as a result of stopping, altering, or reducing the dosage of neuroleptic drugs; this condition is referred to as neuroleptic withdrawal-emergent dyskinesia. Dyskinesia that lasts longer than this time frame is regarded as real TD [9] because withdrawal-emergent dyskinesia is often transient (lasting less than four to eight weeks).

Tetrabenazine is regarded as the first-line treatment for dopamine disorders [9]. Clonazepam and Ginkgo biloba may help treat TD (level B); amantadine and tetrabenazine may also be used to treat TD (level C); however, there is currently inadequate evidence to either support or refute the use of numerous other medications. Alternatively, discontinuing the use of causative agents or transitioning from typical to atypical dopamine receptor antagonists remains uncertain (level U). Branch-chain amino acids are effective in other therapies as well [10,11].

## Conclusions

This is a unique case of Risperidone withdrawal TD that developed after eight months of cessation of the drug. This case report highlights the importance of the incidence of TD in older patients. The patient must be compliant with dopamine-blocking agents, and the physicians should look for the earliest symptom of the onset of TD in patients with multiple risk factors, like in this case, such as old age, prolonged use of Risperidone, and abrupt cessation/non-compliance of the drug. The earliest diagnosis can be treated with a change in medication, altering the dosage of the causative drug. While prescribing antipsychotics, keep in mind that TD can present as a delayed side effect. We recommend further studies to focus on the other drugs that might be responsible for the symptoms of TD in a patient.
